# Sonochemical Synthesis of Ce-doped TiO_2_ Nanostructure: A Visible-Light-Driven Photocatalyst for Degradation of Toluene and O-Xylene

**DOI:** 10.3390/ma12081265

**Published:** 2019-04-17

**Authors:** Joon Yeob Lee, Jeong-Hak Choi

**Affiliations:** 1 Life Environmental R&D Center, CHEMTOPIA Co., Ltd., Seoul 08377, Korea; jlee@chemtopia.net; 2 Department of Environmental Engineering, Catholic University of Pusan, Busan 46252, Korea;

**Keywords:** sonochemical processing method, Ce content, light sources, photocatalyst

## Abstract

Ce-doped TiO_2_ nanostructures (CeT) with different amounts of Ce (0.5, 0.75, 1.0, 1.5, and 2.0 wt. %) were synthesized using a sonochemical processing method. The physicochemical properties of the prepared samples were explored using UV-visible diffuse reflectance spectroscopy (UV-vis DRS), field-emission TEM (FE-TEM), XRD, X-ray photoelectron spectroscopy (XPS), photoluminescence spectroscopy (PL), and surface area and pore size analyzers. The photocatalytic performance of the prepared CeT was assessed by monitoring their degradation efficiencies for gaseous toluene and o-xylene—widely known as significant indoor air pollutants—under daylight irradiation. The prepared CeT exhibited significantly improved photocatalytic performance towards the degradation of toluene and o-xylene, which was much higher than that observed for pure TiO_2_ and commercial P25 TiO_2_. Particularly, photocatalytic degradation efficiencies by the prepared CeT catalysts increased remarkably in the case of o-xylene (up to 99.4%) compared to toluene (up to 49.1%). The degradation efficiency by the CeT was greatest for the CeT-0.75 sample, followed by, in order, CeT-1.0, CeT-0.5, CeT-1.5, and CeT-2.0 samples in agreement with the order of the surface area and the particle size of the catalysts. According to the change of light source, the average decomposition efficiencies for toluene and o-xylene by CeT-0.75 were shown in the order of conventional daylight lamp > violet light emitting diodes (LEDs) > white LEDs. The decomposition efficiencies normalized to supplied electric power, however, were estimated to be in the following order of violet LEDs > white LEDs > conventional daylight lamp, indicating that the LEDs could be a much more energy efficient light source for the photodecomposition of target toluene and o-xylene using the CeT-0.75 photocatalyst.

## 1. Introduction

Aromatic volatile compounds are considered to be serious indoor air pollutants because they can cause adverse health risks for building occupants [1]. The appearance of these compounds indoors results mainly from the wide use of them as building interior materials, household products, and so on [1]. Most aromatic volatile compounds are known for their carcinogenic effects, and in some cases, they can seriously damage the functioning of kidneys, liver, the respiratory system, and the central nervous system of human beings [2]. Actually, it has been reported that the central nervous system (CNS) is likely to be damaged by the exposure to toluene and xylene [3]. Exposure to low to moderate levels of toluene vapor can cause dizziness, nausea, confusion, memory loss, weakness, loss of appetite, hearing, and color vision loss. Long-term exposure to toluene vapors may lead to damage of the central nervous, the reproductive, and the immune systems, and can even cause death [3,4]. Inhaling xylene vapor can also lead to nausea, dizziness, headache, vomiting, loss of consciousness, damage of the central nervous system, and death [5]. Moreover, because a lot of people spend most of their time in indoor environments, the concentration level of these indoor air pollutants should be reduced to minimize the health risks of building occupants.

During the last few decades, titanium dioxide (TiO_2_) has received particular attention in the field of the photocatalytic elimination of various air and water contaminants [6,7,8] due to its effective properties such as non-toxicity, low cost, high resistance to photo-corrosion, and good physical and chemical stabilities [9,10,11]. However, bare TiO_2_ has several drawbacks, including a wide band gap (~3.2 eV), which induces it as active only under UV light illumination (λ < 380 nm) [12]—the undesirable recombination of photoexcited charge carriers in TiO_2_ that results in a low photo quantum yield [11,12,13]. Thus, it is necessary to tune the band structure of TiO_2_ to exhibit satisfactory photocatalytic reactivity under a broad range of solar spectrum including visible range as well as UV range. Moreover, reducing the frequency of the recombination of photoexcited electrons and holes is also essential to improving the photocatalytic performance of TiO_2_. Many strategies have attempted to address the mentioned drawbacks of TiO_2_, including dye sensitization, semiconductor compounding, and ion-doping [14,15,16,17]. In general, doping or incorporation of metal-ions into the TiO_2_ structure is considered to be one of the highly applicable ways to develop novel TiO_2_ catalysts [18]. In principle, the incorporation of metal ions can decrease the band-gap energy and therefore allow the photocatalytic activity of TiO_2_ to expand to visible range [19,20,21]. Additionally, the metal dopants can capture the photoexcited electrons and hamper the charge-carrier recombination by accelerating charge transfer processes [22,23,24]. For these reasons, it is expected that the metal-doped TiO_2_ can work more effectively in decomposing environmental pollutants than the pure TiO_2_ [25,26].

In general, rare earth metal ions are known to be ideal dopants for tuning the electronic and crystal structures and light-harvesting characteristics of TiO_2_ compared to various other metal ions because of the interaction between their f-orbital and the functional groups of organic compounds resulting in the formation of complexes with numerous Lewis bases such as thiols, alcohols, aldehydes, and organic acids [27,28]. Among the available rare earth metal ions, relatively non-toxic and inexpensive cerium (Ce) [29] has attracted more interest as a dopant because of the following reasons: (1) the distinct electronic structures between Ce^4+^ (4f^0^5d^0^) and Ce^3+^ (4f^1^5d^0^) can result in different optical and catalytic properties; (2) the redox couple Ce^3+^/Ce^4+^ leads cerium oxide to shift between CeO_2_ and Ce_2_O_3_ under oxidizing and reducing conditions [30,31].

Additionally, various methods including sol-gel [32], hydrothermal [33], microwave [34], and sonochemical [35] were employed to prepare titania nanomaterials. Recently, ultrasonic synthesis has been used to prepare a variety of nano-structured catalysts in an aqueous solution, including TiO_2_ [35], WO_3_ [36], and ZnO [37]. A powerful driving force for this technique is transiently formed thermodynamics of hotspots during the formation/collapse of the ultrasonic cavitation bubbles, which reach a temperature of about 5000–25,000 K, a high pressure of about 20 Mpa, and heating/cooling rates above 1010 K s^−1^ [35,36,37]. Therefore, high temperature and pressures can lead to changes in the morphology of nanoparticles during ultrasonic treatment.

Therefore, in this study, Ce-doped TiO_2_ (CeT) nanostructures with different amounts of Ce metal ions were synthesized via a sonochemical method known as an excellent and facile route for the fabrication of diverse nano-structured materials [38,39,40]. The fabricated samples were characterized and assessed for the degradation of gaseous toluene and o-xylene under daylight irradiation. The photocatalytic performance of the synthesized CeT was also compared with pure TiO_2_ (PT) and commercial Degussa TiO_2_ (P25). Moreover, two types of light emitting diodes (LEDs) were tested as light sources to compare the effects of the light source on the photodegradation performance, and the energy efficiencies of them were also evaluated.

## 2. Materials and Methods

### 2.1. Preparation of Pure TiO_2_ and Ce-doped TiO_2_ Nanostructures

The Ce-doped TiO_2_ photocatalyst was synthesized by an ultrasonic treatment followed by a calcination process. Cerium nitrate hexahydrate (99%, Sigma–Aldrich, Saint Louis, MO, USA) was used as a source of Ce metal. The different amounts of Ce metal sources determined to provide metal to TiO_2_ weight ratios of 0.5%, 0.75%, 1.0%, 1.5%, and 2.0%, respectively (denoted here as CeT-x, where x = Ce to TiO_2_ wt. %) and 0.5 g of surfactant P-123 (Sigma–Aldrich) were dissolved in deionized water (100 mL) under vigorous stirring. Then, 25 mL of titanium tetraisopropoxide (99.9%, Sigma–Aldrich) was added dropwise into the above solution for about 3 min with magnetic stirring. The resulting solution was subjected to ultrasonic treatment for 60 min (Sonics & Materials VCX750, operating at an amplitude of 29 µm and a frequency of 20 kHz) and was then aged for 12 h. Subsequently, the obtained product in the mixture was separated by centrifugation (5000 rpm), washed with water, and then dried at 80 °C for 12 h in an electric dry oven. The final powder was calcined in an electric furnace at 500 °C for 1 h to get the CeT powder. Bare TiO_2_ was also prepared using this procedure without adding Ce metal.

### 2.2. Characterization

The properties of the prepared photocatalysts were characterized using XRD (D/max-2500 diffractometer, Rigaku Corp., Tokyo, Japan), XPS (Quantera SXM, ULVAC-PHI, Inc., Chigasaki, Japan), UV-vis diffuse reflectance spectroscopy (UV-vis DRS) (CARY 5G, Varian, Palo Alto, CA, USA), field-emission TEM (FE-TEM) (Titan G2 ChemiSTEM (Cs probe), FEI Company, Hillsboro, OR, USA), photoluminescence spectroscopy (PL) (SpectraPro 2150i, Acton Research, Lakewood Ranch, FL, USA), and surface area and pore size analyzers (Autosorb-iQ & Quadrasorb SI, Quantachrome Instruments, Boynton Beach, FL, USA).

### 2.3. Photocatalytic Reactor System and Degradation of Target Compounds

**Figure 1:**
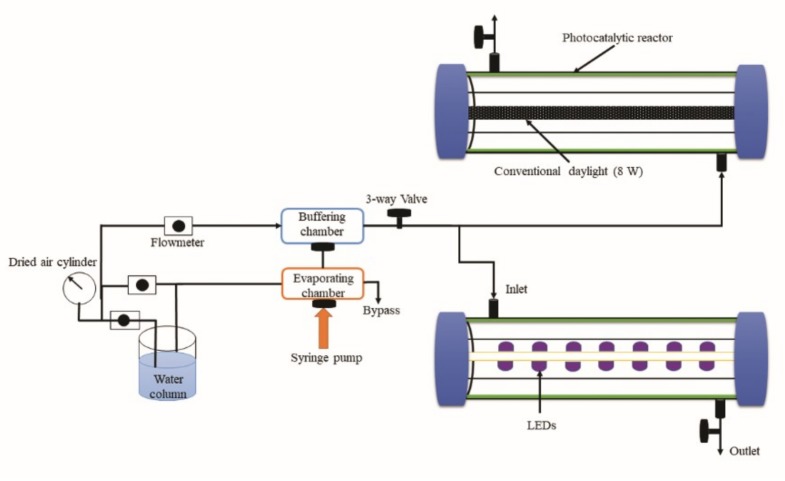
Schematic diagram of photocatalytic reactor system.

The photocatalytic degradation experiments were conducted using a homemade reactor system similar to that used in our previous work [41], which is displayed in Figure 1. Major component parts of the reactor system included a high purity air cylinder for air supply, a water bath for moisture supply, a thermal chamber for mixing of air and standard substrates, a syringe pump for automatic injection of model toluene and o-xylene gases, gas flowmeters for stream flow rates measurement, a 3-way control valve for sampling gaseous target compounds, and a photoreactor (Pyrex tube with an inner diameter of 3.8 cm and a volume of 133 cm^3^) with an 8 W conventional daylight lamp (λ = 400–700 nm) or 0.32 W white (λ = 450 nm) or 0.32 W violet (λ = 400 nm) LEDs as a light source. A spinning method was applied for a thin film coating to the inside wall of the Pyrex reactor with each of the prepared photocatalysts. The high-purity air was passed through the water bath for moisture supply at a specified relative humidity. The humidified air was fed into the photocatalytic reactor perpendicularly in order to increase the mass transport of incoming gas to the photocatalyst surface. The representative operating conditions of the photocatalytic reactor system could be described as follows: gas flow rate, 1.0 L min^−1^; relative humidity, 45 ± 5%; input concentration of gaseous target compounds, 0.1 ppm.

Prior to the photodegradation test, the photoreactor was flushed with high-purity air overnight to remove any other chemicals attached to the system. In the absence of catalyst, the reactor was subjected to light irradiation to explore the impact of light on the decomposition of model pollutants. Adsorption equilibrium between the catalyst and the volatile organic compounds was examined by measuring the concentrations of the compounds in input and output airflow. After reaching the adsorption equilibrium (which meant that the input and output concentrations were equal), the light sources were switched on to start the actual photocatalytic tests.

The quantitative analysis of the model pollutants was determined using a gas chromatograph-mass spectrometer (GC-MS; QP2020Ultra, Shimadzu, Kyoto, Japan). During the test, air samples were collected by drawing air from the sampling ports to Tenax GC-contained stainless steel thermal desorption (TD) tube to concentrate the target compounds. An automatic thermal desorption unit (TD-20, Shimadzu, Japan) was used to transfer sampled compounds to a GC-MS. The adsorbent tube was heat treated, and the chemical species were concentrated on an internal trap. Finally, the internal trap was thermally treated to transport the chemical species to the analytical system.

## 3. Results and Discussion

### 3.1. Characterization of the Fabricated Catalysts

**Figure 2:**
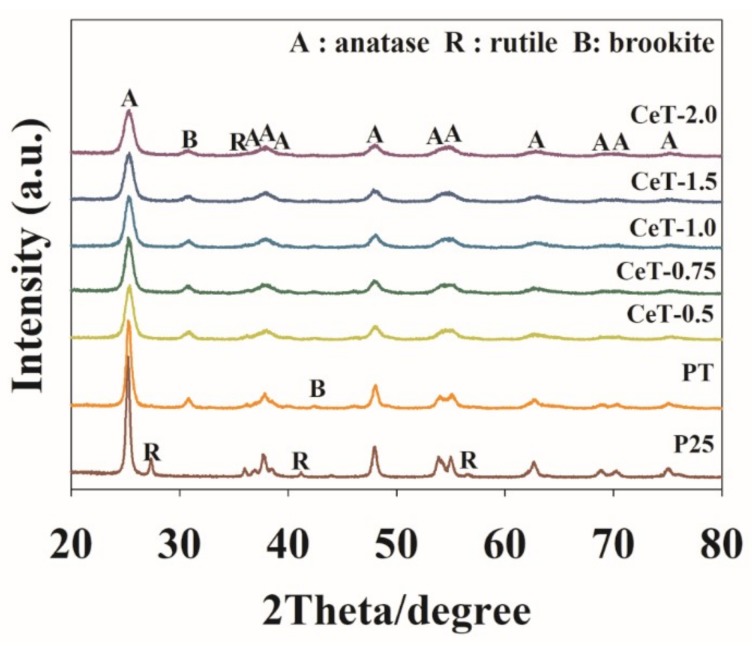
XRD spectra of Degussa P25 TiO_2_ (P25), pure TiO_2_ (PT), and Ce-doped TiO_2_ catalysts with different Ce contents (CeT-0.5, 0.75, 1.0, 1.5, and 2.0).

Figure 2 shows the XRD patterns of P25, PT, and Ce-doped TiO_2_ samples synthesized with five different Ce weight ratios (CeT-0.5, CeT-0.75, CeT-1.0, CeT-1.5, and CeT-2.0). All the photocatalysts used in this study revealed anatase, rutile, and brookite phase structures, and anatase phase peak was exhibited as the main structure with a main peak at 2*θ* = 25.3°. Feng et al. [38] reported slightly different results than the present work where both PT and CeT exhibited two phase structures of anatase and brookite at ratios of 0.892 and 0.108 in PT and 0.782 and 0.218 in CeT, respectively. The observed difference may have been due to differences in synthesis conditions, including ultrasonic treatment time and amplitude as well as calcination temperature. Rutile phase peaks were shown to be relatively smaller in CeT than in P25. The decreased rutile phase in Ce-doped TiO_2_ photocatalysts was due to the fact that the incorporation of Ce metal ions into TiO_2_ lattice may have impeded the transformation of brookite or anatase to the rutile phase [42]. It is also worth noting that with increase in the weight ration of Ce in CeT samples, the peak intensities of the anatase phase slightly declined, and the width of the diffraction peaks at 25.3° became broader, clearly indicating the depletion of the crystallization and the decrease of the crystallite sizes [43]. A shift in the diffraction peaks toward higher 2*θ* was also noticed for the CeT catalysts, which may have demonstrated the shrinkage of some of the TiO_2_ lattices caused by incorporation of the doped Ce.

**Figure 3:**
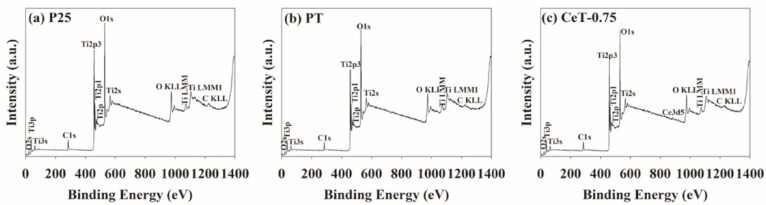
X-ray photoelectron spectroscopy (XPS) profiles of (**a**) Degussa P25 TiO_2_ (P25), (**b**) pure TiO_2_ (PT), and (**c**) Ce-doped TiO_2_ (CeT-0.75).

As shown in Figure 3, XPS analysis was conducted to survey the surface elements in P25, PT, and CeT-0.75 catalysts. Notably, Figure 3c clearly confirms the successful incorporation of Ce metal ions into the TiO_2_ lattice. The peak at 884 eV was attributed to Ce 3d_5/2_, which revealed that Ce existed mainly in the +3 oxidation state (Ce^3+^). The peak intensity of Ce element was very weak due to its low content. The XPS profiles of P25 and PT (Figure 3a,b) showed the only peaks corresponding to Ti 2p_3/2_ (458 eV) and Ti 2p_1/2_ (464 eV), and no other peaks related to Ce were observed.

**Figure 4:**
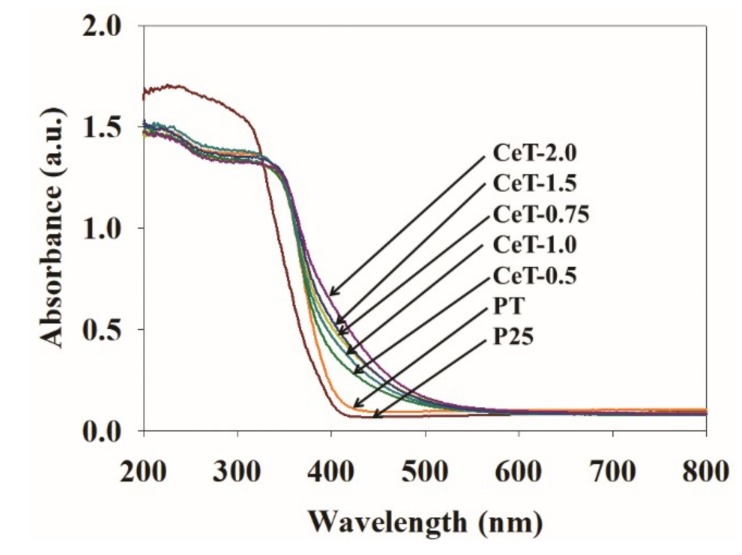
UV-vis diffuse reflectance spectroscopy (UV-vis DRS) of Degussa P25 TiO_2_ (P25), pure TiO_2_ (PT), and Ce-doped TiO_2_ catalysts with different Ce contents (CeT-0.5, 0.75, 1.0, 1.5, and 2.0).

Figure 4 shows the UV-vis DRS patterns of the fabricated photocatalysts. The band-gap energy (Eg) of the fabricated samples could be calculated from UV-vis spectroscopy by using the Equation (1):

Eg = 1240/λ,(1)

where λ is the wavelength (nm). Commercial Degussa P25 TiO_2_ and pure TiO_2_ exhibited a sharp absorbance edge at about 387 nm (3.20 eV) and 398 nm (3.11 eV) and a relatively higher absorbance in the UV region than the other synthesized photocatalysts with no significant absorbance in visible range. This result may have been due to the high rutile phase ratio in P25, as shown in Figure 2. Moreover, the band gaps of P25 and PT were significantly different. It is usually due to the differences in particle size or agglomeration state [44]. Nevertheless, the absorbance edge of all Ce-doped TiO_2_ samples with different Ce weight ratios (CeT-0.5, CeT-0.75, CeT-1.0, CeT-1.5, and CeT-2.0) shifted toward the visible region. This result was in accordance with the previous report [38]. Moreover, the shift of the absorbance edge to the visible-light region increased gradually with the increase in Ce weight ratio of 0.5–2.0 wt. %. The band gap energies of CeT-0.5, CeT-0.75, CeT-1.0, CeT-1.5, and CeT-2.0 samples estimated from their absorbance edges were 3.07 eV (404 nm), 3.03 eV (409 nm), 3.00 eV (413 nm), 2.97 eV (417 nm), and 2.89 eV (429 nm), respectively. The extended absorption into the visible range was likely due to the reduction of the band gap resulting from the increased charge transfer owing to the impurity levels that formed in the TiO_2_ band structure by doped Ce metal ions [25,45]. From these results, it was inferred that the Ce-doped TiO_2_ photocatalyst could be activated effectively under daylight irradiation.

**Figure 5:**
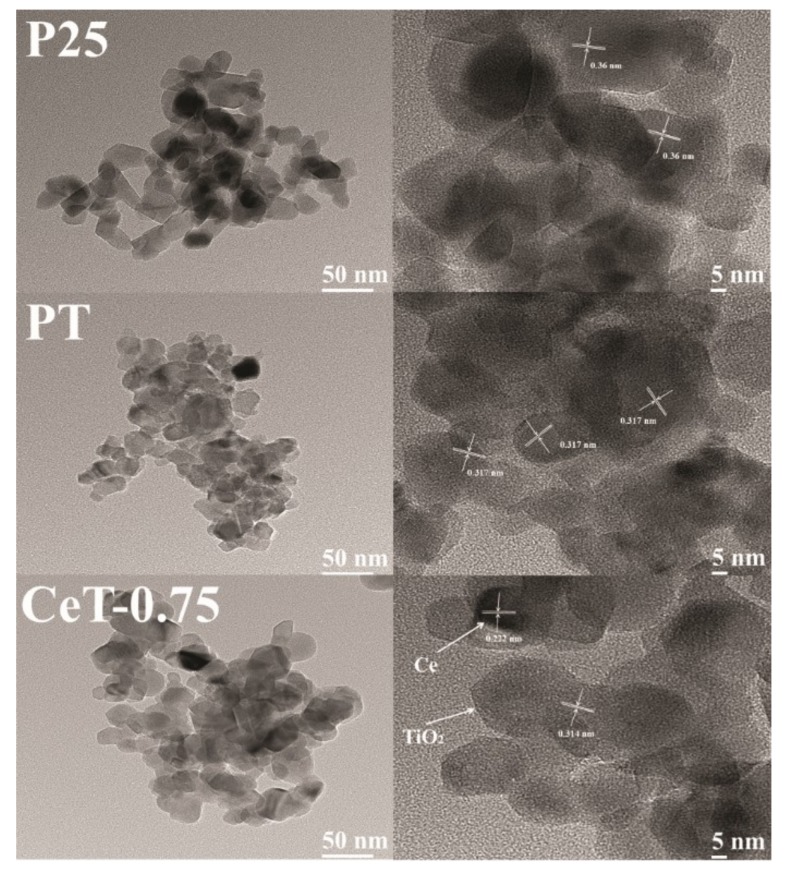
TEM and high-resolution TEM images of Degussa P25 TiO_2_ (P25), pure TiO_2_ (PT), and Ce-doped TiO_2_ (CeT-0.75).

**Figure 6:**
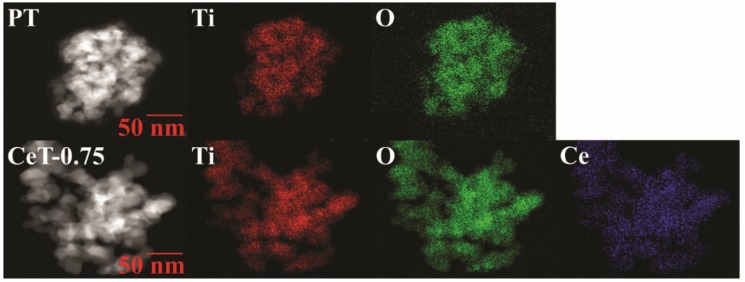
Elemental mapping images of pure TiO_2_ (PT) and Ce-doped TiO_2_ (CeT-0.75) nanostructures.

The FE-TEM images revealed the morphologies and sizes of the selected photocatalysts (i.e., P25, PT, and CeT-0.75), as shown in Figure 5. The images of P25 and PT showed lattice fringes of TiO_2_ with interplanar spacings of 0.36 and 0.317 nm, respectively. On the other hand, CeT-0.75 exhibited the lattice fringes of TiO_2_ and Ce with interplanar spacings of 0.314 and 0.222 nm, respectively. In addition, the elemental mapping images of PT and CeT-0.75 (Figure 6) showed that CeT-0.75 contained Ti, O, and Ce, while PT consisted only of Ti and O. These results indicated that the Ce metal was successfully embedded into the TiO_2_ nanostructures during the synthesis of the CeT photocatalysts.

The average particle sizes of P25, PT, and CeT-0.75 were 24.6 nm, 22.5 nm, and 13.2 nm, respectively, and the other CeT photocatalysts with Ce contents were in the particle size range of 13.6 to 15.3 nm (Table 1). The average particle size was determined using the following Equation (2), assuming all particles to have the same spherical shape and size [46].

*D* = 6000/(*S*_BET_ × *ρ*) (2)

where *D* is the particle size, *S*_BET_ is the Brunauer-Emmett-Teller (BET)-specific surface area, and *ρ* is the true density (*ρ* for titania is 4.2 g mL^−1^).

The CeT-0.75 sample showed the smallest particle size, whereas the commercial P25 showed the largest particle size. Specific surface areas and total pore volumes of the synthesized photocatalysts were also measured, and the results are summarized in Table 1. The CeT-0.75 exhibited the largest surface area of 108.6 m^2^ g^−1^ and pore volume of 0.314 cm^3^ g^−1^, while the P25 exhibited the lowest values of 58.1 m^2^ g^−1^ and 0.198 cm^3^ g^−1^, respectively, in contrast with the results of particle sizes. Additionally, the specific surface areas of all the synthesized CeT photocatalysts were greater than that of pure TiO_2_, and the surface area values were in the order of CeT-0.75 > CeT-1.0 > CeT-0.5 > CeT-1.5 > CeT-2.0. From the results, it could be concluded that the Ce weight ratio of 0.75 wt. % was the optimum catalyst synthesized under the present experimental conditions.

### 3.2. Photocatalytic Performace

**Figure 7:**
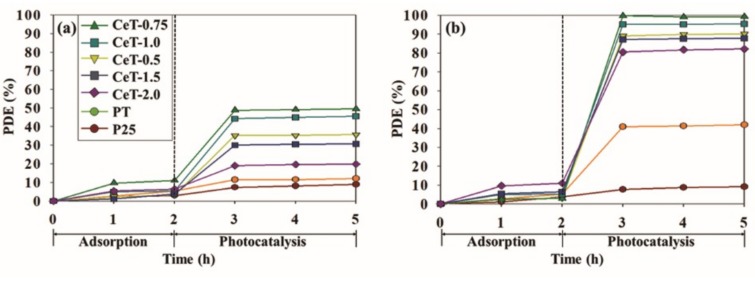
Photocatalytic decomposition efficiencies (PDE, %) for (**a**) toluene and (**b**) o-xylene determined with Degussa P25 TiO_2_ (P25), pure TiO_2_ (PT), and Ce-doped TiO_2_ catalysts with different Ce contents (CeT-0.5, 0.75, 1.0, 1.5, and 2.0).

The photocatalytic activities of the synthesized photocatalysts were investigated by determining the decomposition efficiencies for toluene and o-xylene under daylight irradiation. Adsorption equilibrium between the catalysts and the target toluene and o-xylene pollutants was achieved within 2 h after injecting the gases. Figure 7 displays the photodegradation efficiencies for toluene and o-xylene using P25, PT, and CeT with five different Ce loadings over a 3-h photocatalytic reaction. Over a 3-h photocatalytic reaction, the photocatalytic degradation of the target compounds was almost completed within an initial 1 h, and then there was no noticeable increase in the degradation efficiencies. The degradation efficiencies for the target compounds via CeT photocatalysts were higher than those achieved for P25 and PT. Notably, it was observed that the decomposition efficiencies using CeT photocatalysts increased remarkably in the case of o-xylene compared to toluene. The average decomposition efficiencies for toluene and o-xylene via commercial P25 were 8.1% and 8.5%, respectively. Meanwhile, little increased efficiencies of 11.6% (for toluene) and 41.4% (for o-xylene) were shown in the use of PT. The corresponding degradation efficiencies of five CeT samples (i.e., CeT-0.5, CeT-0.75, CeT-1.0, CeT-1.5, and CeT-2.0) were 35.4%, 49.1%, 44.9%, 30.4%, and 19.4% for toluene and 89.7%, 99.4%, 95.3%, 87.6%, and 81.5% for o-xylene, respectively. It was worth noting that the photocatalytic degradation efficiencies for both target compounds via CeT photocatalysts were determined in the order of CeT-0.75 > CeT-1.0 > CeT-0.5 > CeT-1.5 > CeT-2.0. These results were in agreement with the order of surface area, particle size, and crystallite size of anatase of the five synthesized CeT photocatalysts, as shown in Table 1. Although CeT-0.75 showed less redshift in its light absorption (Figure 4) and wider band gap (Table 1) than CeT-1.5 and CeT-2.0, it yielded the highest photocatalytic reactivity among the prepared photocatalysts, likely because of its increased adsorption capacity. Consequently, it could be described that the photodegradation reactivity of the synthesized photocatalyst was more strongly influenced by its particle size and surface area rather than the redshift or the band gap [38].

The kinetic analysis for the removal of toluene and o-xylene by means of adsorption and photocatalytic degradation was conducted, and the results are shown in Table 2. The following reaction rate equation (Equation (3)) was used for the kinetic analysis:

d*C*/d*t* = −*k*C^n,^ (3)

where *C* is the concentration of target compounds (μg L^−1^), *t* is reaction time (h), *k* is a reaction rate constant, and n is the order of reaction. Overall, both the adsorption step and the photocatalytic degradation step were found to be well fitted to the pseudo-second-order kinetic model. In the adsorption step, CeT-0.75 in the case of toluene (*k*_2_ = 0.1639 L μg^−1^ h^−1^) and CeT-2.0 in the case of o-xylene (*k*_2_ = 0.1424 L μg^−1^ h^−1^) showed the highest reaction rate constant values in this study. In the subsequent photocatalytic degradation, CeT-0.75 showed a much higher reaction rate constant in decomposition of both toluene (*k*_2_ = 0.9227 L μg^−1^ h^−1^) and o-xylene (*k*_2_ = 99.755 L μg^−1^ h^−1^), indicating that CeT-0.75 was the most reactive among the photocatalysts used in this study.

A summary of numerous metal-doped TiO_2_ catalysts applied in photocatalytic elimination of toluene and o-xylene are provided in Table 3, which also compares the photocatalytic decomposition efficiency between bare TiO_2_ and metal-doped TiO_2_. Comprehensively, dopants could enhance the performance of bare TiO_2_ in the decomposition of VOCs (volatile organic compounds) under visible light illumination. They narrowed the band gap of TiO_2_ by shifting the light absorption wavelength of TiO_2_ from the UV region to the visible region.

According to the earlier reports, doping of TiO_2_ with metal ions such as Cr, Cu, Fe, Mn, and Zn has enhanced photocatalytic performance for the elimination of environmental contaminants [49,50,51,52]. Some other research groups reported that dual-doped TiO_2_ nanoparticles with non-metal (N) and metal ion (Fe) showed higher performance than those of N doped or pure TiO_2_ on the degradation of 2,4-dichlorophenol and methylene blue under visible light exposure [53,54].

It is well known that the improved photocatalytic performance of the metal-ion doped TiO_2_ is most likely due to three main factors: (1) adsorption capacity, (2) separation of charge carriers, and (3) tuning of band gap [45]. The band gaps of the CeT photocatalysts (2.89–3.07 eV) were lower than those of P25 (3.20 eV) and PT (3.11 eV), as shown in Table 1.

**Figure 8:**
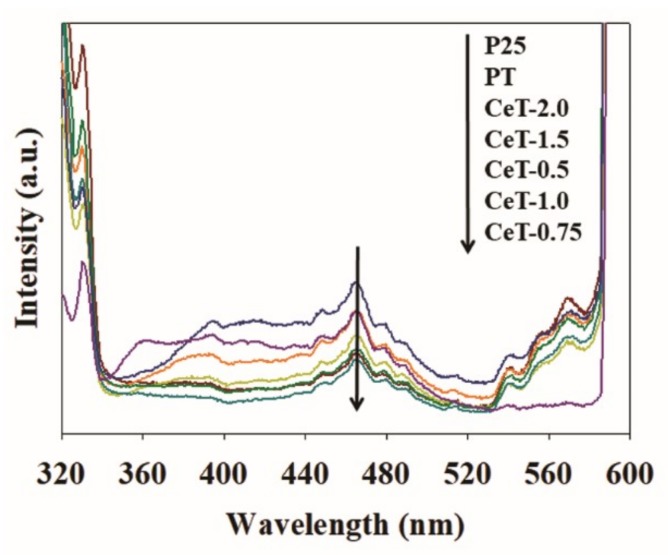
Photoluminescence spectroscopy (PL) profiles of Degussa P25 TiO_2_ (P25), pure TiO_2_ (PT), and Ce-doped TiO_2_ catalysts with different Ce contents (CeT-0.5, 0.75, 1.0, 1.5, and 2.0).

The band gap narrowing was ascribed to the metal incorporation that created new electronic states in the band structure, allowing enhanced photocatalytic performances under visible light irradiation. Moreover, the embedded metal ions could capture the electrons, the enhancement of interfacial charge transfer, and the reduction of the recombination of photoexcited charge carriers. The embedded metal elements into the TiO_2_ photocatalyst would also improve the separation of photoexcited electron-hole pairs. This was supported by the PL spectra (Figure 8), which displayed weak PL emission intensities of CeT photocatalysts compared to those of P25 and PT. This clearly suggested that the separation efficiencies of the CeT catalysts were higher than those of the P25 and PT samples, since the PL emission intensity was proportional to the recombination of charge carriers [55]. The PL emission intensities of the CeT samples were also determined in the order of CeT-2.0 > CeT-1.5 > CeT-0.5 > CeT-1.0 > CeT-0.75, indicating that the CeT-0.75 had the weakest PL emission intensity among all the catalysts studied (Figure 8).

**Figure 9:**
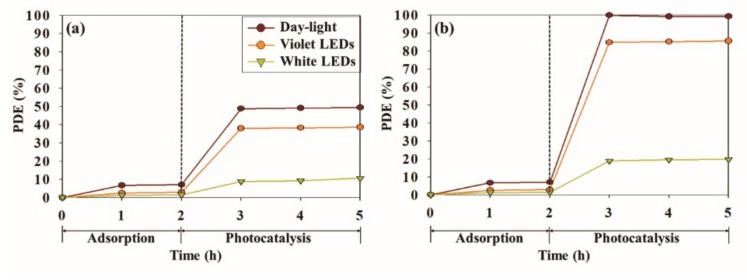
Photocatalytic decomposition efficiencies (PDE, %) for (**a**) toluene and (**b**) o-xylene determined with CeT-0.75 under three types of light sources (conventional daylight lamp, violet light emitting diodes (LEDs), and white LEDs).

Figure 9 depicts the photodecomposition efficiencies for toluene and o-xylene using the CeT-0.75 photocatalyst with the three types of light sources—the 8 W conventional daylight lamp, the 0.32 W white LEDs, and the 0.32 W violet LEDs. After achieving the adsorption equilibrium between the CeT-0.75 catalyst and the target compounds in a dark condition over 2 h, the light irradiation started for the photocatalytic reaction between the photocatalyst and the target chemicals. Over a 3-h photocatalytic reaction, the photocatalytic degradation efficiencies increased within an initial 1 h, and then no conspicuous increment in the degradation efficiencies was shown. In addition, the decomposition efficiencies for o-xylene were evaluated as relatively high compared to those for toluene. The average photodecomposition efficiencies for toluene and o-xylene with the CeT-0.75 photocatalyst according to the light sources were shown in the order of conventional daylight lamp > violet LEDs > white LEDs: 49.1 and 99.4% for conventional daylight lamp, respectively; 40.0 and 85.1% for violet LEDs, respectively; 9.2 and 19.4% for white LEDs, respectively. However, the photodecomposition efficiencies normalized to supplied electric powers were estimated to be in the order of violet LEDs > white LEDs > conventional daylight lamp: 1.25 and 2.66%/W for violet LEDs, respectively; 0.28 and 0.60%/W for white LEDs, respectively; 0.06 and 0.12%/W for conventional daylight lamp, respectively. From these results, it could be interred that the 0.32 W LEDs were much more energy-efficient light sources than the 8 W conventional daylight lamp for the photodecomposition of toluene and o-xylene with the CeT-0.75 photocatalyst, although the photocatalytic reactivity was shown to be higher under the daylight irradiation.

A possible photocatalytic mechanism for the degradation of various organic compounds using metal-doped TiO_2_ catalysts was described in previous studies [38,42]. Under light irradiation onto the metal-doped TiO_2_ catalysts, a photoexcited electron was transferred from the valence band of TiO_2_ to newly created electronic states in the band structure by metals incorporated into the TiO_2_ lattice [18,22,23]. Subsequently, the transferred electron reacted with an oxygen molecule (O_2_) to generate a superoxide radical anion (∙O_2_^−^), and the positive hole in the valence band of the TiO_2_ reacted with a hydroxyl ion (OH^−^) or a water molecule (H_2_O) to form a hydroxyl radical (∙OH). After that, the reactive ∙O_2_^−^ and ∙OH could oxidize the target organic compounds to generate CO_2_, CO, and other byproducts. Consequently, the enhanced performance of the Ce-doped TiO_2_ photocatalyst for the photodecomposition of toluene and o-xylene under the visible-light illumination could also be ascribed to the above-mentioned reaction mechanism. In this study, according to Lee and Jo [41], the reaction mechanism could be modified as follows:

Ce-doped TiO_2_ + visible light → e^−^ + h^+^, (4)

O_2_ + e^−^ → ∙O_2_^−^, (5)

∙O_2_^−^ + toluene and o-xylene → CO_2_ + gas- and liquid-phase intermediates, (6)

OH^−^ + h^+^ → ∙OH, (7)

∙OH + toluene and o-xylene → CO_2_ + gas- and liquid-phase intermediates (8)

## 4. Conclusions

Ce-doped TiO_2_ nanostructures were successfully fabricated by a simplified sonochemical process that could extend the absorbance spectra to a visible range and reduce the frequency of the recombination of photoexcited charge carriers. It was also found that the amount of Ce doping could influence the morphology and the surface properties of the prepared samples, such as particle size, specific surface area, and crystallite size of anatase. The Ce weight ratio of 0.75% was the optimal Ce doping amount in this study. Compared with bare TiO_2_ and commercial P25 TiO_2_, the photocatalytic performance of the prepared Ce-doped TiO_2_ for the decomposition of toluene and o-xylene under daylight illumination was obviously improved. In particular, the photocatalytic degradation efficiencies by the prepared Ce-doped TiO_2_ increased conspicuously in the case of o-xylene compared to the toluene. The degradation efficiencies by the Ce-doped TiO_2_ samples were shown in the order of CeT-0.75 > CeT-1.0 > CeT-0.5 > CeT-1.5 > CeT-2.0, in agreement with the order of the surface area and the particle size of the samples. While a conventional daylight lamp showed the highest decomposition efficiency among the used three types of light sources, violet LEDs could be a far more energy efficient light source for the photodecomposition of toluene and o-xylene by CeT-0.75. Consequently, it could be concluded that Ce-doped TiO_2_ nanostructures synthesized via a simplified sonochemical process might have good potential for application in the control of indoor air pollutants since they showed an efficient visible light photoactivity in the decomposition of gaseous toluene and o-xylene.

## Figures and Tables

**Table 1:** Properties of Degussa P25 TiO_2_ (P25), pure TiO_2_ (PT), and Ce-doped TiO_2_ catalysts with different Ce contents (CeT-0.5, 0.75, 1.0, 1.5, and 2.0).

Photocatalyst	*S*_BET_ (m^2^ g^−1^)	Total Pore Volume (cm^3^ g^−1^)	Particle Size (nm)	Crystallite Size of Anatase (nm)	Band Gap, Eg (eV)
P25	58.1	0.198	24.6	24.2	3.20
PT	63.5	0.212	22.5	16.4	3.11
CeT-0.5	96.7	0.312	14.8	12.4	3.07
CeT-0.75	108.6	0.314	13.2	10.9	3.03
CeT-1.0	104.7	0.312	13.6	11.7	3.00
CeT-1.5	94.5	0.309	15.1	12.7	2.97
CeT-2.0	93.5	0.310	15.3	12.8	2.89

**Table 2:** Kinetic parameters of adsorption and photodegradation of toluene and o-xylene using Degussa P25 TiO_2_ (P25), pure TiO_2_ (PT), and Ce-doped TiO_2_ nanoparticles (CeT-0.5, 0.75, 1.0, 1.5, and 2.0).

Kinetic Model		Pseudo-First-Order Kinetic Model				Pseudo-Second-Order Kinetic Model			
Target Compound		Toluene		o-Xylene		Toluene		o-Xylene	
Parameter		*k*_1_ (h^−1^)	*R* ^2^	*k*_1_ (h^−1^)	*R* ^2^	*k*_2_ (L μg^−1^ h^−1^)	*R* ^2^	*k*_2_ (L μg^−1^ h^−1^)	*R* ^2^
Adsorption step	P25	0.0147	0.8524	0.0189	0.9461	0.0396	0.8537	0.0443	0.9442
	PT	0.0272	1.0000	0.0272	1.0000	0.0742	1.0000	0.0645	1.0000
	CeT-0.5	0.0168	0.9525	0.0168	0.9525	0.0453	0.9542	0.0393	0.9542
	CeT-0.75	0.0583	0.8484	0.0147	0.8524	0.1639	0.8532	0.0344	0.8537
	CeT-1.0	0.0272	0.7934	0.0272	0.7934	0.0742	0.7945	0.0645	0.7945
	CeT-1.5	0.0189	0.9461	0.0331	0.8669	0.0510	0.9442	0.0788	0.8700
	CeT-2.0	0.0331	0.8669	0.0583	0.8484	0.0907	0.8700	0.1424	0.8532
Photocatalytic degradation step	P25	0.0326	0.9447	0.0338	0.9167	0.0905	0.9466	0.0816	0.9178
	PT	0.0453	0.9159	0.1937	0.9045	0.1280	0.9173	0.5925	0.9078
	CeT-0.5	0.1573	0.9003	0.8274	0.8998	0.5241	0.9020	7.5242	0.9101
	CeT-0.75	0.2429	0.8975	1.7160	0.9057	0.9227	0.8992	99.755	0.9475
	CeT-1.0	0.2160	0.9024	1.0981	0.8966	0.7871	0.9055	16.990	0.9046
	CeT-1.5	0.1304	0.8950	0.7508	0.8949	0.4173	0.8954	5.9113	0.8978
	CeT-2.0	0.0786	0.8989	0.6144	0.9014	0.2333	0.8995	3.7749	0.9103

**Table 3:** Comparison of the photodegradation efficiencies of VOCs (volatile organic compounds) with literature values.

Dopant	Doped TiO_2_ Preparation Method	Target Compounds	Initial Pollutant Concentration (ppm)	Light Sources	Photocatalytic Degradation Efficiency (%)		Ref.
					Doped TiO_2_	Pure TiO_2_	
Fe	Solvothermal method	Toluene	1000–1200	Visible light	48	20	[47]
W					54		
V					69		
Fe	Electrospinning method	Benzene, Toluene, Ethylbenzene, and o-Xylene	0.1	Visible light	68 (toluene) and 91 (o-xylene)	35 (toluene) and 58 (o-xylene)	[48]
Ce	Sonochemical method	Toluene and o-Xylene	0.1	Visible light	49 (toluene) and 99.4 (o-xylene)	11.6 (toluene) and 41.4 (o-xylene)	This study
